# Intelligent spectropolarimetry

**DOI:** 10.1038/s41377-024-01493-3

**Published:** 2024-07-09

**Authors:** Fakun Wang, Qi Jie Wang

**Affiliations:** School of Electrical & Electronic Engineering & School of Physical and Mathematical Sciences, Nanyang Technological University, Singapore, Singapore

**Keywords:** Optical materials and structures, Optical techniques

*Nature* **630**, 77–83 (2024) 10.1038/s41586-024-07398-w

High-dimensional polarization and spectral information are crucial in a wide range of applications, from everyday life to national security. However, obtaining this complex information typically requires cumbersome systems composed of separate polarimeters and spectrometers. To address this challenge, a team of researchers from the Changchun Institute of Optics, Fine Mechanics and Physics, Chinese Academy of Sciences, University of Chinese Academy of Sciences, National University of Singapore, and Australian National University has developed an innovative intelligent spectropolarimetry approach. This method employs a uniform dispersive thin-film architecture that modulates polarization and spectral responses in the wavevector domain, allowing the capture of high-dimensional information in a single snapshot image. Deep residual networks then facilitate the efficient decoding of this complex data. Remarkably, this technique enables the simultaneous examination of mixed full-Stokes polarization states and broadband spectral information using just one device and a single measurement. Seamlessly integrating with existing imaging platforms, this advancement offers a compact yet powerful solution for high-dimensional photodetection and imaging.

